# Correlation between Intraocular Pressure Fluctuation with Postural Change and Postoperative Intraocular Pressure in Relation to the Time Course after Trabeculectomy

**DOI:** 10.1155/2014/801967

**Published:** 2014-07-17

**Authors:** Kazuyuki Hirooka, Kaori Tenkumo, Eri Nitta, Shino Sato

**Affiliations:** Department of Ophthalmology, Kagawa University Faculty of Medicine, 1750-1 Ikenobe, Miki, Kagawa 761-0793, Japan

## Abstract

*Background.* To investigate the correlation between intraocular pressure (IOP) fluctuation with postural change and IOP in relation to the time course after trabeculectomy. *Methods.* A total of 29 patients who had previously undergone primary trabeculectomy with mitomycin C were examined. IOP was obtained at 1, 2, 3, 6, and 12 months and then every 6 months postoperatively. *Results.* The postural IOP difference before surgery was 3.0 ± 1.8 mmHg, which was reduced to 0.9 ± 1.1 mmHg at 1 month, 1.0 ± 1.0 mmHg at 2 months, 1.3 ± 2.0 mmHg at 3 months, 1.3 ± 1.4 mmHg at 6 months, 1.4 ± 1.5 mmHg at 12 months, and 1.1 ± 0.7 mmHg at 18 months after trabeculectomy (*P* < 0.01 each visit). The filtering surgery failed in 7 out of 29 eyes. Postural IOP changes were less than 3 mmHg in those patients who did not require needle revision at every visit. However, in patients who did require needle revision, the increase in the posture-induced IOP was greater than 3 mmHg prior to the increase in the sitting position IOP. *Conclusions.* Assessment of postural IOP changes after trabeculectomy might be potentially useful for predicting IOP changes after trabeculectomy.

## 1. Introduction

Intraocular pressure (IOP) is a major risk factor for the development of glaucoma. Changes in body position have been shown to affect IOP [1–6]. It is generally believed that postural changes have a greater effect on the IOP in glaucomatous eyes than in normal eyes [3, 6–8]. In addition to the supine position being an important contributor to the IOP elevation observed at night, this position has also been shown to be associated with the progression of open-angle glaucoma [6, 9, 10]. Thus, when treating glaucoma patients, it is very important to be able to ascertain the IOP changes that occur due to postural changes. Several topical hypotensive eye drops with a variety of pharmacologic mechanisms are currently available. However, timolol maleate, latanoprost, and brinzolamide do not decrease the magnitude of the IOP elevation associated with postural change [11].

Trabeculectomy is the recommended surgical therapy for eyes with glaucoma, as this procedure leads to a greater IOP reduction than other therapeutic interventions. Furthermore, it has been reported that eyes that have undergone trabeculectomy have less IOP fluctuation during the diurnal IOP changes than medically treated glaucoma eyes [12, 13]. Some investigators have additionally reported that trabeculectomy not only decreases the IOP but also reduces the degree of the posture-induced IOP changes [14–16]. However, when the bleb function fails, it has been shown that the postural IOP changes return to baseline levels [16].

The purpose of the current study was to investigate the correlation between the sitting IOP and the degree of postural IOP changes in relation to the time course after trabeculectomy.

## 2. Materials and Methods

Between August 2011 and August 2012, this prospective, consecutive series examined 30 eyes of 30 open-angle glaucoma patients who were treated with trabeculectomy and followed up at Kagawa University Hospital, Kagawa, Japan. Patients with a history of intraocular surgery, including laser trabeculoplasty, were excluded. Clinical characteristics of the studied subjects are listed in Table 1. Males and females, aged 50 to 87 years, were eligible for this study. The study group consisted of 21 primary open-angle glaucoma (POAG) and 3 normal-tension glaucoma (NTG) patients, with each patient treated with a topical hypotensive agent in both eyes. All patients underwent visual acuity, refraction, central and peripheral field, slit-lamp, and gonioscopy examinations. Glaucomatous eyes were defined as eyes exhibiting structural glaucomatous changes (vertical cup-disc asymmetry between fellow eyes of ≥0.2, a cup-to-disc ratio of ≥0.6, and neuroretinal rim narrowing, notches, localized pallor, or retinal nerve fiber layer defects with glaucomatous visual field (VF) loss in the corresponding hemifield). A glaucomatous VF was defined as a glaucoma hemifield test outside normal limits on at least two consecutive baseline tests and the presence of at least three contiguous test points within the same hemifield on the pattern deviation plot at *P* < 1%, with at least one at *P* < 0.5% when excluding points on the edge of the field or those directly above and below the blind spot. Patients with an IOP ≥ 22 mmHg were diagnosed as POAG while NTG was diagnosed when the untreated peak IOP was ≤21 mmHg, which included 24-hour fluctuations. All eligible subjects received a detailed explanation before both the trabeculectomy and the study. All patients signed an informed consent form in accordance with the principles embodied in the Declaration of Helsinki. This study was approved by the Institutional Review Board of the Kagawa University Hospital.

Throughout the experimental period, a single examiner used the ICare rebound tonometer (ICare; Tiolat Oy, Helsinki, Finland) to measure the IOP in the morning for each of the patients before the trabeculectomy and at 1, 2, 3, 6, and 12 months and then every 6 months postoperatively. During the actual measurements, each subject was asked to sit comfortably at the edge of an examining bed in a quiet room. All IOPs were measured with the ICare rebound tonometer while the patients remained in a sitting position. After this reading, patients were asked to lie on a bed and then turn to a lateral decubitus position with their heads placed on a soft pillow. The body was positioned so that the eye scheduled for the surgery was located directly above the other eye. Body position was maintained for 5 minutes. IOP was measured in that position with the ICare tonometer. After the examiner asked the patients to gaze straight ahead and look at a fixation point, the IOP measurement was made by touching the transducer to the center of the patients' cornea. Three consecutive sets of measurements (six measurements for each set) were made. The average of each set was automatically calculated, with the averaged values used for the statistical analysis.

All of the patients were examined approximately 6 times/year. Patient visits tended to be more frequent during the early postoperative period. At each visit, all patients underwent a standard ophthalmologic examination that included slit-lamp, Goldmann applanation tonometry, and binocular fundus examinations. To maintain good control of the IOP, laser suture lysis (performed within 1 month after trabeculectomy) was used when necessary. Cases of serious intra- or postoperative complications were excluded from the study.

Statistical analyses were performed using SPSS version 19.0 (IBM, New York, NY). IOP differences before and after the trabeculectomy were compared using a paired *t*-test. IOP differences between the operated eye and the nonoperated other eye were evaluated by an unpaired *t*-test. A *P* value less than 0.05 was considered statistically significant. Data are presented as the mean ± standard deviation.

## 3. Results

Out of the 30 subjects examined, 29 eyes of 29 patients completed the protocol. The one patient who did not complete the study developed serious hypotonic maculopathy at 3 months after the filtering surgery. Preoperative demographic data are shown in Table 1.

**Figure 1:**
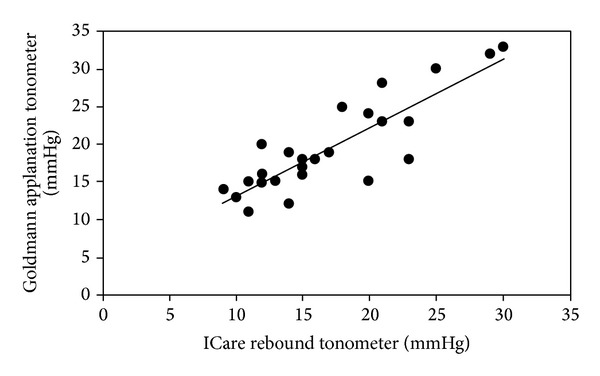
Comparative IOP using Goldmann applanation tonometer and ICare for seated patients. Correlation coefficient = 0.86. Regression equation *Y* = 0.91*x* + 3.96.

Figure 1 shows the correlation between the Goldmann applanation tonometer and ICare on subjects in the sitting position at baseline. Both were in close agreement with a correlation coefficient of 0.86 (*P* < 0.001).

**Figure 2:**
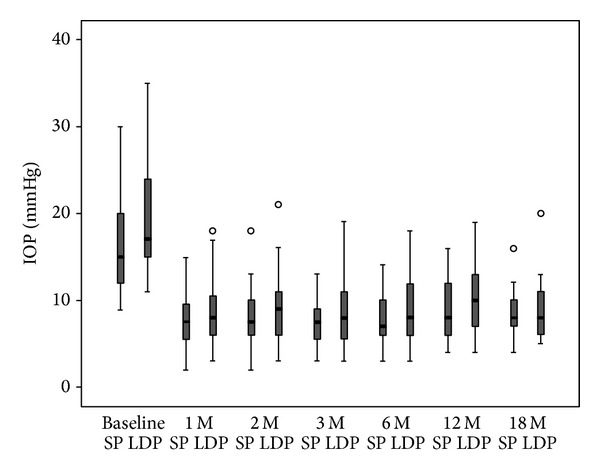
Posture-induced intraocular pressure (IOP) changes measured with an ICare rebound tonometer before and after trabeculectomy over an 18-month period. The IOPs in both the sitting and the lateral decubitus positions were decreased at every visit after the trabeculectomy (*P* < 0.01 every visit; Bonferroni test). The IOP in the lateral decubitus position was significantly increased (*P* < 0.05; paired *t*-test). The squares, upper bars, and lower bars indicate 25–75%, 95%, and 5% percentiles, respectively.

At baseline, the mean IOP in the sitting position was 16.7 ± 5.5 mmHg (range; 9–30 mmHg) (Figure 2). After assuming the lateral decubitus position, the IOP increased in all patients. Prior to surgery, the IOP in the lateral decubitus position was significantly higher than that in the sitting position (*P* < 0.001). The mean change in the IOP between the two body positions was 3.0 ± 1.6 mmHg. One month after trabeculectomy, there were significant reductions of the IOP in the sitting position to 7.8 ± 3.2 mmHg (range; 2–15 mmHg) and in the lateral decubitus position to 8.6 ± 3.9 mmHg (range; 3–18 mmHg) (Figure 2). The difference in the IOP between the sitting and lateral decubitus positions was statistically significant (*P* < 0.001; paired *t*-test). Postoperative changes in the IOP for the sitting and lateral decubitus positions were 7.8 ± 3.3 mmHg and 8.8 ± 4.1 mmHg at 2 months (*P* < 0.001; paired *t*-test), 8.1 ± 3.6 mmHg and 9.4 ± 5.3 mmHg at 3 months (*P* = 0.002; paired *t*-test), 7.7 ± 2.8 mmHg and 9.0 ± 3.6 mmHg at 6 months (*P* < 0.001; paired *t*-test), 8.9 ± 3.2 mmHg and 10.3 ± 4.1 mmHg at 12 months (*P* < 0.001; paired *t*-test), and 8.3 ± 2.8 mmHg and 9.1 ± 3.6 mmHg at 18 months (*P* = 0.03; paired *t*-test), respectively. The degree of reduction of the posture-induced IOPs relative to the difference before the surgery was significant at 1, 2, 3, 6, 12, and 18 months postoperatively (*P* < 0.001, *P* < 0.001, *P* = 0.001, *P* < 0.001, *P* = 0.002, and *P* = 0.001, resp.; Bonferroni test).

Needle revision was required in seven cases (more than 15 mmHg in the sitting position). In 22 patients who did not require needle revisions at every visit, the postural IOP changes were less than 3 mmHg. None of the patients required the administration of any topical hypotensive agents.  Table 2 shows both the sitting position IOPs and the postural IOP changes in the patients who required needle revision. All of these patients initially showed increased posture-induced IOP changes (greater than 3 mmHg), after which increased IOPs were then observed in the sitting position. The mean change in the IOP between the two body positions was 0.9 ± 0.6 mmHg (range; 0–2 mmHg) in the success group and 5.0 ± 1.2 mmHg (range; 4–7 mmHg) in the failure group. The difference in the change in the mean postural-induced IOP between the two groups was statistically significant (*P* < 0.001, unpaired *t*-test).

## 4. Discussion

The results of this study confirm earlier reports [14–16] that trabeculectomy not only reduces the IOP in the sitting position but also reduces the degree of the posture-induced changes in the IOP. The main finding of our current study was that the measurement of postural IOP changes might be a beneficial method for assessing whether a filtering bleb is functioning.

There has been a lot of speculation on the potential mechanism responsible for the postural IOP rise. Although some investigators have hypothesized that it is due to choroidal vascular congestion and increased episcleral venous pressure [17, 18], it has also been suggested that it might be unrelated to aqueous production [19]. The findings for the posture-induced IOP changes following trabeculectomy have been contradictory. Anderson and Grant [1] and Parsley et al. [20] reported that the IOP changes that occurred when moving from a sitting to supine position were greater following glaucoma surgery than in nonoperated, medically treated glaucomatous eyes. However, we have recently reported that after trabeculectomy there was a decrease not only for the IOP in the sitting position but also for the magnitude of the IOP elevation associated with the postural change [14]. Weizer et al. [15] found that the posture-induced IOP change was significantly lower in trabeculectomized eyes than in the nonoperated contralateral eyes. Sawada and Yamamoto [16] showed that trabeculectomy not only decreased the IOP but also reduced the degree of the posture-induced IOP changes if patients had a cystic filtering bleb. However, they also found that if the bleb function failed, the postural IOP changes returned to baseline levels. Some investigators have suggested that the posture-induced IOP decrease after trabeculectomy might mainly be due to the ability of the trabeculectomy to lower the IOP [15, 21]. However, Sawada and Yamamoto [22] recently reported that eyes that underwent successful trabeculectomy had smaller posture-induced IOP changes as compared to nonoperated, medically treated eyes, with the IOP in both groups found to be 12 mmHg or less. Trabeculectomy creates a new aqueous pathway via the filtering bleb that is independent of the episcleral veins. Therefore, it seems reasonable that trabeculectomy could cause a suppression of the posture-induced IOP alternations.

Prior to surgery, the mean change in the IOP between the two body positions was 3.0 ± 1.6 mmHg. Therefore, we determined 3 mmHg as an optimal cut-off value. The most important finding in our current study was that there was an increase in the posture-induced IOP prior to the increase in the IOP in the sitting position. Posture-induced IOP changes have been shown to rapidly occur within 5 minutes after a position change [23, 24]. When the filtering bleb function is working, posture-induced IOP changes are suppressed within 5 minutes after changing the position. However, when the filtering bleb function worsens, it appears that more time is required (at least more than 5 minutes) in order to reduce or fail to reduce the posture-induced IOP changes, even when the IOP in the sitting position is still low enough. Furthermore, when there is continued worsening of the filtering bleb function, not only posture-induced IOP but also the IOP in the sitting position increases.

Although the use of 5-fluorouracil (5-FU) and mitomycin *C* has been shown to improve the outcome of filtration surgeries, additional treatments are commonly required in order to manage failed filtration blebs. Several reports have shown needle revision to be an effective and relatively simple method for reestablishing filtration in eyes with failed filtration blebs [25–27]. However, failure of the initial 5-FU needling revision has been shown to be significantly correlated with higher preneedling IOP [26, 28]. Thus, it has been recommended that the needle revision be performed as soon as there is an increase in the IOP or detection of bleb flattening. If it were possible to predict a failing filtering bleb before the IOP begins to increase, then simple monitoring of patients followed by a quick reaction to any changes in the IOP after the surgical procedure could lead to a greatly improved success rate for needle revision.

The limitations of our present work include having only a short follow-up period and a small sample size. A second limitation was that we did not evaluate the inner structure of the filtering bleb using ultrasound biomicroscopy or anterior segment optical coherence tomography.

## 5. Conclusions

In conclusion, assessment of postural IOP changes after trabeculectomy might be potentially useful in predicting IOP changes in the sitting position after trabeculectomy. However, further long-term followups and large-scale investigations will need to be undertaken in order to definitively clarify this issue.

## Figures and Tables

**Table 1:** Participant demographic data.

Age (y)	66.9 ± 11.0
Gender (male/female)	20/9
Diagnosis	
POAG	21
NTG	8
IOP with GAT (mmHg)	19.2 ± 5.8

POAG: primary open-angle glaucoma.

NTG: normal-tension glaucoma.

IOP: intraocular pressure.

GAT: Goldmann applanation tonometer.

**Table 2:** Individual data for sitting and postural IOP changes.

Patient	Age (yrs)	Gender	Diagnosis	Sitting IOP (ΔIOP)							
				Baseline	After surgery						
					1 m	2 m	3 m	6 m	12 m	18 m	24 m
1	83	M	POAG	12 (2)	11 (1)	13 (3)	16 (4)				
2	51	M	POAG	17 (7)	15 (3)	18 (3)	21 (7)				
3	74	M	POAG	12 (2)	6 (0)	8 (1)	9 (0)	10 (3)	12 (4)	16 (4)	
4	75	M	POAG	30 (5)	9 (1)	11 (1)	8 (0)	8 (2)	14 (4)	19 (5)	
5	50	M	NTG	14 (3)	14 (3)	10 (3)	11 (3)	14 (4)	16 (4)		
6	60	F	NTG	15 (3)	8 (2)	10 (1)	13 (6)	18 (6)			
7	77	F	POAG	21 (5)	10 (1)	7 (1)	7 (1)	5 (2)	8 (4)	10 (3)	17 (5)

IOP: intraocular pressure, ΔIOP: difference between the sitting and lateral decubitus IOP, m: month, M: male, F: female, POAG: primary open-angle glaucoma, and NTG: normal-tension glaucoma.
